# SDOCT Thickness Measurements of Various Retinal Layers in Patients with Autosomal Dominant Optic Atrophy due to *OPA1* Mutations

**DOI:** 10.1155/2013/121398

**Published:** 2013-08-19

**Authors:** Andrea M. Schild, Tina Ristau, Julia Fricke, Antje Neugebauer, Bernd Kirchhof, Srinivas R. Sadda, Sandra Liakopoulos

**Affiliations:** ^1^Department of Ophthalmology, University Hospital of Cologne, 50924 Cologne, Germany; ^2^Cologne Image Reading Center, Department of Ophthalmology, University Hospital of Cologne, 50924 Cologne, Germany; ^3^Doheny Image Reading Center, Doheny Eye Institute, 1450 San Pablo Street, Los Angeles, CA 90033, USA

## Abstract

*Purpose*. To specify thickness values of various retinal layers on macular spectral domain Optical Coherence Tomography (SDOCT) scans in patients with autosomal dominant optic atrophy (ADOA) compared to healthy controls. *Methods*. SDOCT volume scans of 7 patients with ADOA (OPA-1 mutation) and 14 healthy controls were quantitatively analyzed using manual grading software. Mean thickness values for the ETDRS grid subfields 5–8 were calculated for the spaces neurosensory retina, retinal nerve fiber layer (RNFL), ganglion cell layer (GCL), a combined space of inner plexiform layer/outer plexiform layer/inner nuclear layer (IPL+INL+OPL), and a combined space of outer nuclear layer/photoreceptor layers (ONL+PL). *Results*. ADOA patients showed statistically significant lower retinal thickness values than controls (*P* < 0.01). RNFL (*P* < 0.001) and GCL thicknesses (*P* < 0.001) were significantly lower in ADOA patients. There was no difference in IPL+INL+OPL and in ONL+PL thickness. *Conclusion*. Manual subanalysis of macular SDOCT volume scans allowed detailed subanalysis of various retinal layers. Not only RNFL but also GCL thicknesses are reduced in the macular area of ADOA patients whereas subjacent layers are not involved. Together with clinical findings, macular SDOCT helps to identify patients with suspicion for hereditary optic neuropathy before genetic analysis confirms the diagnosis.

## 1. Introduction

Autosomal dominant optic atrophy (ADOA; OMIM 165500) is caused by a mutation in the *OPA1* gene  [1, 2]. The *OPA1* gene encodes a dynamin-related GTPase that plays an important role in mitochondrial fusion processes, stabilization of cristae, and cytochrome c sequestration  [3, 4]. ADOA generally presents with mild visual impairment in the first decade of life. Over years, ADOA leads to a slow continuous decrease in visual acuity (VA), which varies among individuals. Finally, visual loss ranges from a very mild affection to severe impairment  [5, 6].

Visual fields usually show central, paracentral, or centrocecal scotomas. Atrophy of the optic disc may not be visible in the beginning, but with progression of the disease the optic disc typically shows a temporal or diffuse pallor [7]. Histological examinations have revealed a loss in retinal ganglion cells especially in the macular area and in the papillomacular bundle [8]. Loss in vision has been demonstrated to correspond to a reduction of the retinal nerve fiber layer (RNFL) on Stratus optical coherence tomography (OCT) [9]. A reduction of RNFL and GCL thicknesses is also seen in patients with optic atrophy of other origin [10, 11].

Spectral domain OCT (SDOCT) instruments allow improved visualization of various retinal layers with almost histological quality. The technology of SDOCT is noninvasive, fast, and easy to apply even in young children  [12].

The aim of this study was to analyze thickness values of various retinal layers on SDOCT volume scans of the posterior pole using manual OCT grading software in patients with ADOA associated with *OPA1* mutations compared to healthy controls.

## 2. Materials and Methods

Seven right eyes of 7 unrelated patients with genetically confirmed ADOA with heterozygous mutation in the *OPA1* gene were compared to 14 right eyes of 14 healthy controls matching in age, refractive error, and gender.

SDOCT (Spectralis OCT, Heidelberg Engineering, Germany) derived volume scans of the macular area (15 × 20°) and best corrected VA were retrospectively collected for patients and controls. Volume scans were quantitatively analyzed using computer-assisted manual grading software (3D-OCTOR), which facilitates manual delineation of various retinal layers. The software has been validated in previous reports  [13–15]. After drawing the required layers for all spaces with a computer mouse, the software generates thickness and volume values for each of the nine subfields of the Early Treatment of Diabetic Retinopathy Study (ETDRS) grid.

**Figure 1:**
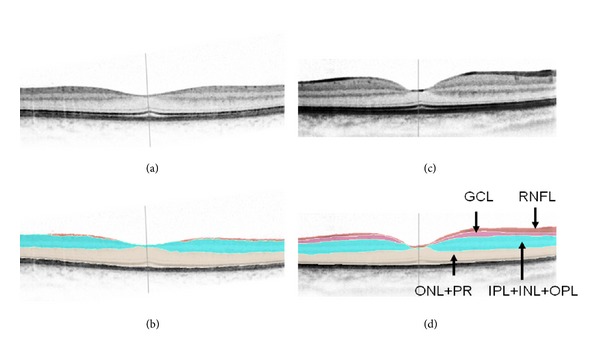
SDOCT scans with and without delineation of retinal boundaries for neurosensory retina, retinal nerve fiber layer (RNFL), ganglion cell layer (GCL), a combined space of inner and outer plexiform layers and inner nuclear layer (IPL+INL+OPL), and a combined space of the outer nuclear layer and photoreceptor layer (ONL+PR): A+B: ADOA patient, C+D: healthy control.

**Figure 2:**
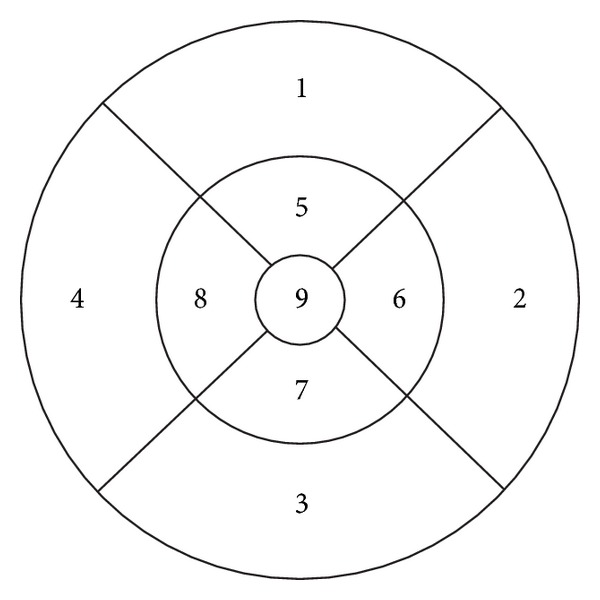
SDOCT thickness map with EDTRS inner circle (5–8) for OD: subfield 8: temporal, subfield 6: nasal.

After the ETDRS grid was manually centered on the fovea, mean thickness values for the inner circle of the ETDRS grid (fields 5–8, 3 mm diameter) were calculated for the following spaces: neurosensory retina, retinal nerve fiber layer (RNFL), ganglion cell layer (GCL), the combined space of inner and outer plexiform layers and inner nuclear layer (IPL+INL+OPL), and the combined space of outer nuclear layer and photoreceptor layer (ONL+PL) (Figures 1 and 2). The foveal central subfield (FCS, 1 mm diameter) was not included in the analysis due to the variability of the foveal depression, which may influence thickness measurements.

Statistical analysis was performed using commercially available software (Sigma Plot for Windows version 11.0 Systat Software Inc., Germany). The Mann-Whitney *U* Test was used to compare thickness values between patients and controls. Intraindividual thickness values of the subfields 5 to 8 were compared using ANOVA on ranks analysis. For statistical analysis, Snellen VA was converted to logarithm of the minimum angle of resolution (logMAR). The correlation between OCT parameters and VA was assessed using Spearman's rank correlation coefficient and regression analysis. This study adhered to the tenets set forth in the Declaration of Helsinki.

## 3. Results

Four male and 3 female ADOA patients were included in the study. The mean age was 25 years (min 4; max 47). VA ranged from 20/800 to 20/40. Two patients reported progressive visual loss since early childhood. In 5 patients, the diagnosis was made by chance during a routine check by an outpatient ophthalmologist. Clinical and genetic data of all patients are summarized in Table 1.

Fourteen right eyes of 14 healthy individuals were included in the study as controls. The mean age was 27 years (min 3; max 54). Refractive error ranged from −6.0 dpt to +2.3 dpt (spherical equivalent) comparable to the group of patients. VA was 20/20 in all cases. Visual fields and disc appearance were unremarkable. A summary of median thickness values of all subfields is provided in Table 2.

Median thickness of the neurosensory retina was statistically significant lower in ADOA patients compared to healthy controls (*P* < 0.01). Subanalysis of retinal layers revealed that RNFL thickness (*P* < 0.001) as well as GCL thickness (*P* < 0.001) measurements were significantly lower in patients with ADOA for each of the ETDRS subfields 5, 6, 7, and 8. However, there was no difference in ONL+PR thickness values as well as in IPL+INL+OPL thickness values between groups. The differences in thickness values between patients and controls were not only found in adult patients but also in the two children of 4 and 11 years of age with recent diagnosis of the disease.

Intraindividual subanalysis of the mean thickness values for subfields 5 to 8 revealed significant difference between the nasal, temporal, inferior, and superior subfields in both groups. In ADOA patients, RNFL thickness appeared to show the lowest values in subfield 8 (temporal) and a statistically significant difference was detected only for RNFL thickness of subfield 8 compared to subfield 5 (*P* = 0.04). For all other spaces, there was no difference between subfields.

In healthy controls, neurosensory retina was significantly thinner in subfield 8 than in subfield 5 (*P* < 0.001), subfield 6 (*P* = 0.006), and subfield 7 (0.02), RNFL in subfield 8 was significantly thinner compared to subfield 5 (*P* < 0.001), subfield 6 (*P* < 0.001), and subfield 7 (*P* < 0.001), and subfield 6 was significantly thinner compared to subfield 5 (*P* = 0.01), and subfield 7 (*P* < 0.001). The combined space of INL+OPL+IPL was also significantly thinner in subfield 8 than in subfield 5 (*P* = 0.03), subfield 6 (*P* = 0.01) and subfield 7 (0.02). GCL and ONL+PR showed no difference between subfields.

In our study group of ADOA patients, neither regression analysis nor Spearman's analysis showed a significant correlation between VA or age and GCL thickness or RNFL thickness. 

## 4. Discussion

High resolution SDOCT images and manual subanalysis of retinal layers allowed detailed quantitative analysis of various inner and outer retinal layers within the macular region in ADOA patients. We could confirm that inner retinal layers are primarily affected in ADOA patients and demonstrate that thinning of inner retinal layers is explained by a reduction in RNFL thickness as well as GCL thickness without any effect on IPL+INL+OPL thickness. The results from our study are in line with marked loss of GCL and retinal ganglion cell axons in the macula as previously documented in postmortem histopathological studies of patients with ADOA  [8]. Lower thickness values of inner retinal layers were even evident in young patients newly diagnosed with the disease.

Recent OCT studies mainly focused on measurements of peripapillary RNFL. Milea et al.  [16] reported a gradual reduction in peripapillary RNFL thickness with age in 10 ADOA patients with *OPA1* mutations as well as in 30 healthy subjects using Stratus OCT. In ADOA patients, VA decreased significantly with age. Further, VA was correlated with peripapillary RNFL thickness in the inferior and superior peripapillary quadrants and with total macular thickness at eccentricities of 500–3000 *μ*m. In contrast, we did not neither find a significant correlations between RNFLs at the posterior pole and age nor between RNFLs at the posterior pole and VA in our group. Differences in the individual course of the disease as well as our relatively small study group might be the reason for the lack in significant correlations with VA and age in our study.

Yu-Wai-Man et al. [9] recently also demonstrated a generalized decrease in peripapillary RNFL thickness on Stratus OCT in 40 patients with *OPA1* mutations, with a statistically significant inverse correlation between RNFL thickness and logMAR VA. Additionally, they reported that the temporal quadrant was more severely affected and the nasal quadrant relatively spared, documenting the preferential involvement of the papillomacular bundle in ADOA patients.

These findings were confirmed by Barboni et al.  [17], who compared peripapillary RNFL thickness of 33 patients to healthy controls using Stratus OCT.

Ito et al.  [18] analyzed Stratus OCT images from the peripapillary region in 8 patients with *OPA1* mutations. Additionally, the neurosensory retina was measured at the foveal center point and at two locations 1 and 2 mm temporal, nasal, superior, and inferior to the fovea for the following layers: RNFL, a “middle layer” including the GCL, IPL, INL, and OPL, a layer including the ONL and the photoreceptor inner segments, and the photoreceptor outer segment layer. The neurosensory retina in the macular area was found to be significantly thinner in patients with ADOA compared to healthy subjects at all points measured except for the fovea, and this was explained by a decrease in thickness of the combined GCL, IPL, INL, and OPL in the macular area.

Russo et al.  [19] first used SDOCT images for analysis. Similar to Ito et al., they found a diffuse thinning of the combined space of RNFL, GCL, and IPL in sectional macular SDOCT scans of four affected family members with ADOA. Further, they confirmed the peripapillary RNFL thickness loss and found a correlation between average circumpapillary RNFL and VA.

As the papillomacular bundle was shown to be preferentially involved before  [8, 9, 17], we focused in our study on macular SDOCT volume scans. To allow for a more detailed subanalysis of various retinal layers, we used high-resolution SDOCT images and manual grading software to calculate mean thickness values for various retinal spaces for the ETDRS subfields 5–8. We were able to measure the GCL separately and show that not only the RNFL but also the GCL is significantly reduced in ADOA patients compared to healthy controls, but IPL+INL+OPL thickness is not different to healthy subjects. This difference in RNFL and GCL was evident for the superior, inferior, nasal, and temporal subfield of the ETDRS grid. In healthy controls subfield 8 (temporal) showed the thinnest retinal thickness, INL+IPL+OPL and RNFL values compared to the other subfields. For RNFL, this difference was even evident in ADOA patients in our group.

Beside several strengths, namely, the use of SDOCT and analysis of different retinal layers manually delineated on each *B*-scan, our study also has limitations such as the small study group and the inclusion of children despite the lack of a normative database in the young.

## 5. Conclusions

In conclusion, manual subanalysis of high-resolution macular SDOCT volume scans revealed that not only RNFL thickness but also GCL thickness is reduced in ADOA patients in subfields 5–8, whereas there is no significant difference in IPL+INL+OPL thickness. For patients with loss of vision of unclear origin, SDOCT helps to distinguish between disorders of the outer retinal layers and disorders of the inner retinal layers, for example, optic neuropathies. Together with clinical history and examination, SDOCT may help to identify patients with suspicion for hereditary optic neuropathies before genetic analysis confirms the diagnosis. This technique may further be important for the documentation of possible effects of future therapies like gene therapy, in addition to clinical parameters like measurement of VA and visual fields.

## Figures and Tables

**Table 1:** Clinical and genetic details of ADOA patients.

Case	Gender	*OPA1* mutation	Age at examination (years)	Visual acuity (Snellen) OD/OS	Refraction (spherical equivalent) OD/OS	Visual fields	Disc appearance
1	m	c1299_1305del12bp	4	20/200/20/100	+2.4/+2.4	—	Temporal pallor
2	f	Duplication Exon 1 to 4b of *OPA1* gene	11	20/40/20/125	−0.6/−0.5	Scotomas in the upper temporal fields	Temporal pallor
3	m	c.2614−9A>G	18	20/60/20/60	−2.0/−1.5	Scotomas in the upper fields	Temporal pallor
4	f	c.904A>C, p.Thr302Pro	26	20/100/20/125	−5.0/−5.5	Slight, unspecific defects	Temporal pallor
5	m	c.1979G>Ap.Trp660Stop heterozygous	28	20/60/20/60	+0.5/+1.0	Scotomas OS>OD especially in the upper temporal fields	Temporal pallor
6	m	c.2708_2711 del TTAG p.Val903GlyfsX3 heterozygous	43	20/125/20/100	−2.0/−2.9	Scotomas in the upper fields	Pale disc, especially temporally
7	f	c.2983+4 A>G	47	20/800/20/800	−10.5/−8.3	—	Pale disc, especially temporally

OD: right eye; OS: left eye.

**Table 2:** Median thickness values of retinal spaces in ADOA patients and healthy controls.

Retinal space	Subfield 5		Subfield 6		Subfield 7		Subfield 8		Inner circle	
	(*μ*m)		(*μ*m)		(*μ*m)		(*μ*m)		(*μ*m)	
	Patients	Controls	Patients	Controls	Patients	Controls	Patients	Controls	Patients	Controls
Neurosensory retina	259.7	311.2	259.7	314.0	256.5	312.5	258.4	299.8	259.9	310.1
RNFL	14.2	35.6	13.1	30.6	11.5	35.6	9.1	25.6	10.8	31.3
GCL	0.5	44.9	0.2	48.2	0.2	44.3	0.7	44.8	0.7	45.1
IPL+INL+OPL	124.7	126.7	124.4	124.0	128.1	122.9	123.0	115.0	126.1	123.6
ONL+PL	113.9	111.0	123.5	113.6	116.0	110.3	122.9	117.1	117.7	112.8

RNFL: retinal nerve fiber layer; GCL: ganglion cell layer; INL: inner nuclear layer; IPL: inner plexiform layer; OPL: outer plexiform layer; ONL: outer nuclear layer; PL: photoreceptor layer.
